# Detection of the Presence of Gold Nanoparticles in Organs by Transmission Electron Microscopy

**DOI:** 10.3390/ma3094681

**Published:** 2010-09-27

**Authors:** Wim H. De Jong, Marina C. Burger, Marcel A. Verheijen, Robert E. Geertsma

**Affiliations:** 1 Laboratory for Health Protection Research, National Institute for Public Health and the Environment (RIVM), PO Box 1, 3720 BA Bilthoven, The Netherlands; 2 Laboratory for Infectious Diseases Screening, National Institute for Public Health and the Environment (RIVM), PO Box 1, 3720 BA Bilthoven, The Netherlands; E-Mail: Marina.Burger@rivm.nl; 3 MiPlaza, Philips Research Europe, High Tech Campus 11, 5656 AE Eindhoven, The Netherlands; E-Mail: m.a.verheijen@philips.com; 4 Centre for Biological Medicines and Medical Technology, National Institute for Public Health and the Environment (RIVM), PO Box 1, 3720 BA Bilthoven, The Netherlands; E-Mail: Robert.Geertsma@rivm.nl

**Keywords:** gold nanoparticles, tissue distribution, transmission electron microscopy

## Abstract

Gold nanoparticles of 10 nm and 250 nm were intravenously injected in rats. At 24 h after administration, tissues were collected and prepared for transmission electron microscopy (TEM). In the liver and spleen of animals treated with 10 nm gold nanoparticles, groups of nanoparticles were observed that could be positively identified by Energy Dispersive X-ray (EDX) analysis to contain gold, while nanoparticles could not be detected in the heart, kidney and brain. The 10 nm gold nanoparticles were present in the phagocytic cells of the reticulo-endothelial system (RES). The 250 nm gold nanoparticles could not be detected in any of the organs investigated. Considering the number of 250 nm gold nanoparticles administered, calculations showed that it would indeed be almost impossible to detect the 250 nm gold nanoparticles in TEM preparations in view of the very low number of particles that would be theoretically present in one TEM tissue section. This shows that relatively high numbers of nanoparticles need to be administered to enable the detection of nanoparticles in organs by TEM. In a number of samples, several globular structures of approximately the expected size were found in liver cells and the endothelium of blood vessels in the brain. However, elemental analysis with EDX detection showed that these structures did not contain gold. Our studies thus indicate that the *in vivo* identification of nanoparticles cannot only depend on the detection of nanosized structures in cells. An additional identification of the composing elements of the nanomaterial is necessary for a positive identification of the nanomaterial.

## 1. Introduction

The fast growing number of applications of engineered nanoparticles in drug delivery systems, medical devices, food products, consumer products and the subsequent disposal of engineered nanoparticles in the environment implies that human exposure to engineered nanoparticles is expected to increase greatly. The specific physico-chemical properties at the nanoscale are expected also to result in increased reactivity with biological systems. So, in addition to their beneficial effects, engineered nanoparticles of different types may represent a potential hazard to human health. 

Kinetic properties are considered to be an important descriptor for potential human toxicity and thus for human health risk. It is important to know the amount of the total external exposure that will be absorbed by the body and result in an internal exposure. In addition, the distribution of absorbed nanoparticles in the body over the various organ systems and within the organs needs to be determined. We previously performed a kinetic study to determine the influence of particle size on the *in vivo* tissue distribution of spherical-shaped gold nanoparticles in the rat [1]. Gold nanoparticles were chosen as model substances as they are used in several medical applications. In addition, the detection of the presence of gold is feasible, with no background levels in the body in the normal situation. Rats were intravenously injected in the tail vein with gold nanoparticles with a diameter of 10, 50, 100 or 250 nm. After 24 h, the rats were sacrificed and blood and various organs were collected for gold determination. The presence of gold was measured quantitatively with inductively coupled plasma mass spectrometry (ICP-MS). 

For all gold nanoparticle sizes the majority of the gold was demonstrated to be present in blood, liver and spleen after 24 h [1]. A clear difference was observed between the distribution of the 10 nm particles and the larger particles. The 10 nm particles were present in various organ systems including blood, liver, spleen, kidney, testis, thymus, heart, lung and brain, whereas the larger particles were only detected in the blood, liver and spleen. The results demonstrate that tissue distribution of gold nanoparticles is size-dependent, with the smallest (10 nm) nanoparticles showing the most widespread organ distribution.

For the determination of the gold distribution, the presence of gold was determined by ICP-MS. This gives reliable information on the distribution of the material in the various organ systems, assuming that gold is not naturally present in these organs. However, there is no information on the presence of gold in the form of the actual nanoparticles or their exact location in the organs. One of the methods to identify the actual presence of metal nanoparticles is by transmission electron microscopy (TEM). With this method, local accumulation in cells and the localization of the nanoparticles in cellular organelles can also be investigated. For interpretation of possible toxic effects of nanoparticles, it is important to identify in what type of cells nanoparticles are present, and what the cellular localization of the nanoparticles is. This short communication describes the results of the evaluation of tissue samples with transmission electron microscopy (TEM) for the presence of gold nanoparticles after intravenous administration in rats. 

## 2. Results

### 2.1. TEM Evaluation of Nanoparticle Samples before Injection

**Figure 1:**
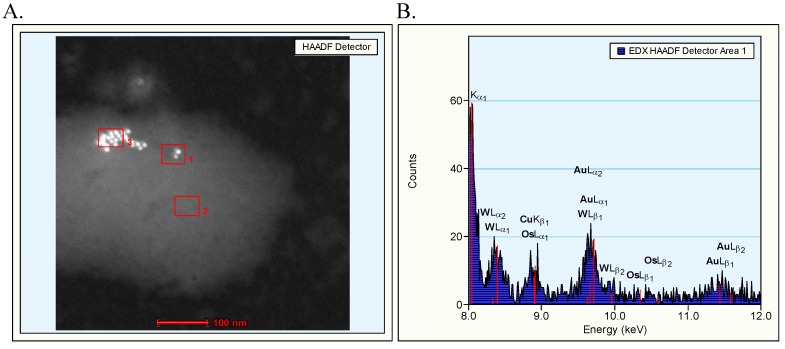

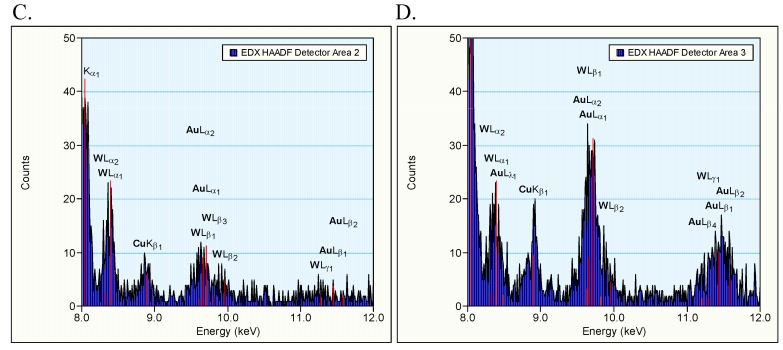
EDX spectrum analysis of 10 nm gold suspension. **A.** The red squares highlight evaluated areas 1–3; **B.** Area 1 shows the presence of two nanoparticles, which at EDX spectrum analysis contained gold; **C.** Area 2 as negative control area contained only wolfram (W); **D.** Area 3 shows an agglomerate of particles identified to contain gold.

**Figure 2:**
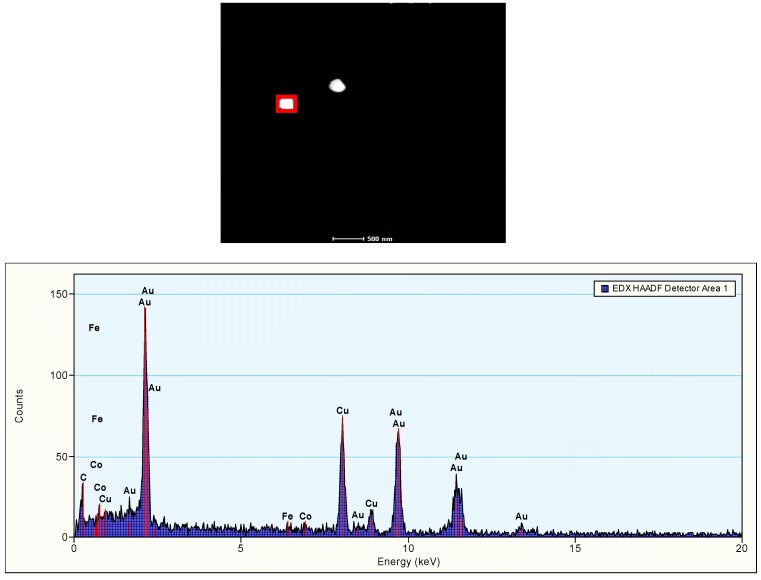
The presence of 250 nm gold nanoparticles in solution. Top: HAADF-STEM image depicting two Au-particles. The red rectangle indicates the region that was scanned during EDX acquisition. Bottom: EDX analysis of single 250 nm gold nanoparticle as present in the solution administered to the animals. In the graph the acquired spectrum of the marked area is presented. It is clearly visible that the particle consists of Au. The other signals in the spectrum can be explained: C, Cu: from the support grid and film, Fe, Co: from the lenses of the microscope.

The TEM evaluation of the gold nanoparticles used in this study has been reported elsewhere [1]. The 10 nm sample showed mainly individual nanoparticles and some clusters, in which up to 60 nanoparticles could be counted. The nanoparticles in the cluster were loosely arranged and individual nanoparticles could be easily recognized, indicating that the clusters consisted of agglomerates with weak binding forces. In samples of nanoparticles of 50, 100 and 250 nm, individual nanoparticles and some clusters of 2–8 nanoparticles were observed. Energy dispersive X-ray (EDX) spectrum analysis demonstrated the presence of 10 nm gold nanoparticles in the solutions (Figure 1). In the spectrum there is a considerable peak overlap between the wolfram (W) and gold (Au) peaks. By comparing relative peak sizes it can be concluded whether the presence of Au is really detected. Although individual gold nanoparticles showed a much weaker signal than groups of nanoparticles, the individual nanoparticles could be identified as consisting of gold. Single 250 nm gold nanoparticles clearly can be positively identified by the EDX spectrum analysis (Figure 2).

### 2.2. TEM Evaluation of Tissue Samples

**Figure 3:**
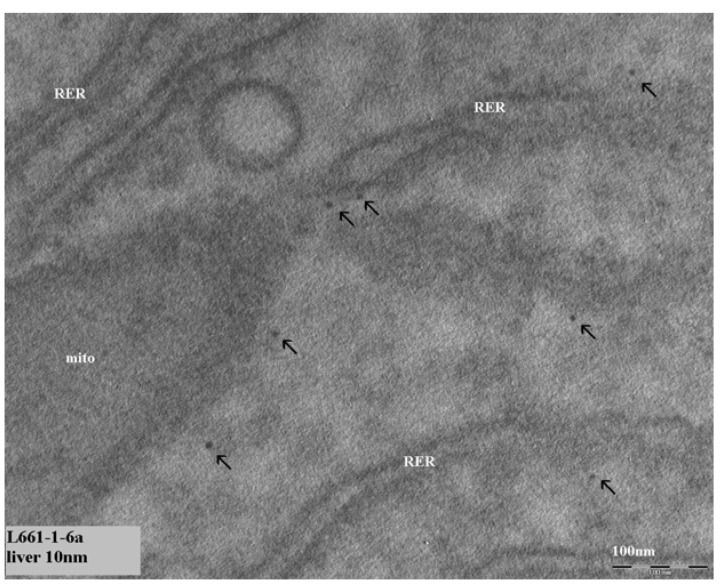
Presence of structures in the cytoplasm of liver cells. Arrows indicate nanosized (8–10 nm) structures in cytoplasm of cells. The marker indicates 100 nm.

**Figure 4:**
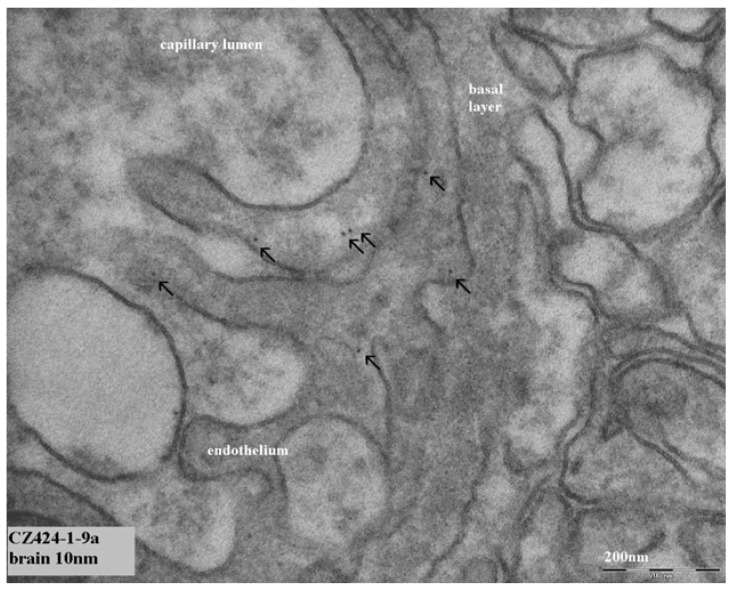
Presence of structures in endothelial cells of the brain. Arrows indicate nanosized (8–10 nm) structures in endothelial cells. The marker indicates 200 nm.

**Figure 5:**
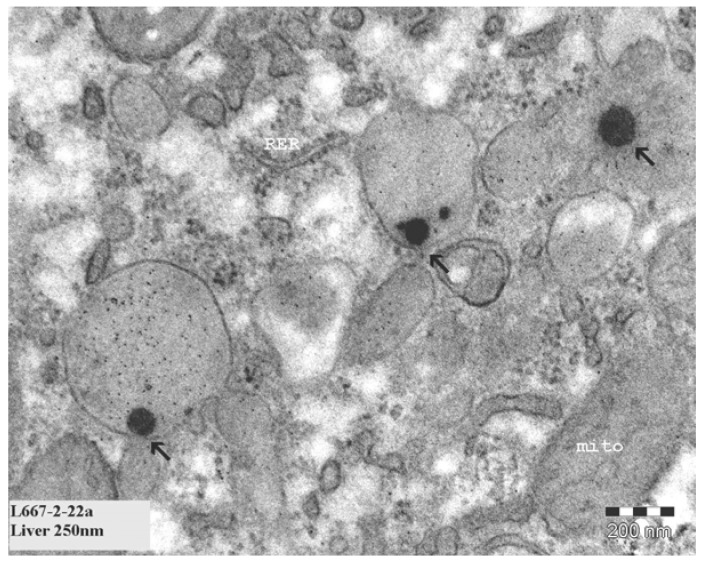
Presence of 100–200 nm structures in liver cells. The marker indicates 200 nm.

For the evaluation of tissue samples, only the tissues were evaluated from animals treated with 10 nm or 250 nm gold nanoparticles as a major difference in tissue distribution was observed between these two nanoparticle sizes [1]. Organs of four animals were evaluated (two animals treated with 10 nm gold nanoparticles and two animals treated with 250 nm gold nanoparticles). The main focus of the TEM evaluation was on liver and spleen as one of the functions of these organs is the removal of agents and particles from the blood circulation. After intravenous administration of the gold nanoparticles, the highest levels of gold were observed in liver and spleen with ICP-MS [1]. Initially, liver and brain (is there really passage of the blood brain barrier?) were evaluated as organs of interest based on the results of our ICP-MS study [1]. Eight to ten nanometer structures were observed in the cytoplasm of most liver cells (hepatocytes) of the animals treated with 10 nm gold nanoparticles. An example of these structures is presented in Figure 3. Also in the brain, these structures were regularly observed in the cytoplasm of the endothelium of the blood vessels (Figure 4). After administration of 250 nm gold nanoparticles, 150–400 nm structures were observed to a lesser extent in the liver cells compared to the 8–10 nm structures. In contrast to the 10 nm structures, these 15–400 nm structures were located in the liver cells in vacuoles surrounded by a membrane, suggesting a lysosome (Figure 5). Comparing the structures observed in the tissue samples with the nanoparticles as previously reported by us [1], a difference in appearance was noted. The gold nanoparticles in dispersion show very sharp edges and a sharp contrast compared to the surroundings, whereas the structures in the tissues showed rather soft borders without a sharp edge and an irregular black intensity. In order to elucidate the identity of these structures, additional TEM studies were performed with EDX spectrum analysis. The observed structures with a diameter of 8–10 nm and the structures with a diameter of 150–400 nm were found not to contain gold. So, these structures initially observed were not the gold nanoparticles injected in these animals. 

**Figure 6:**
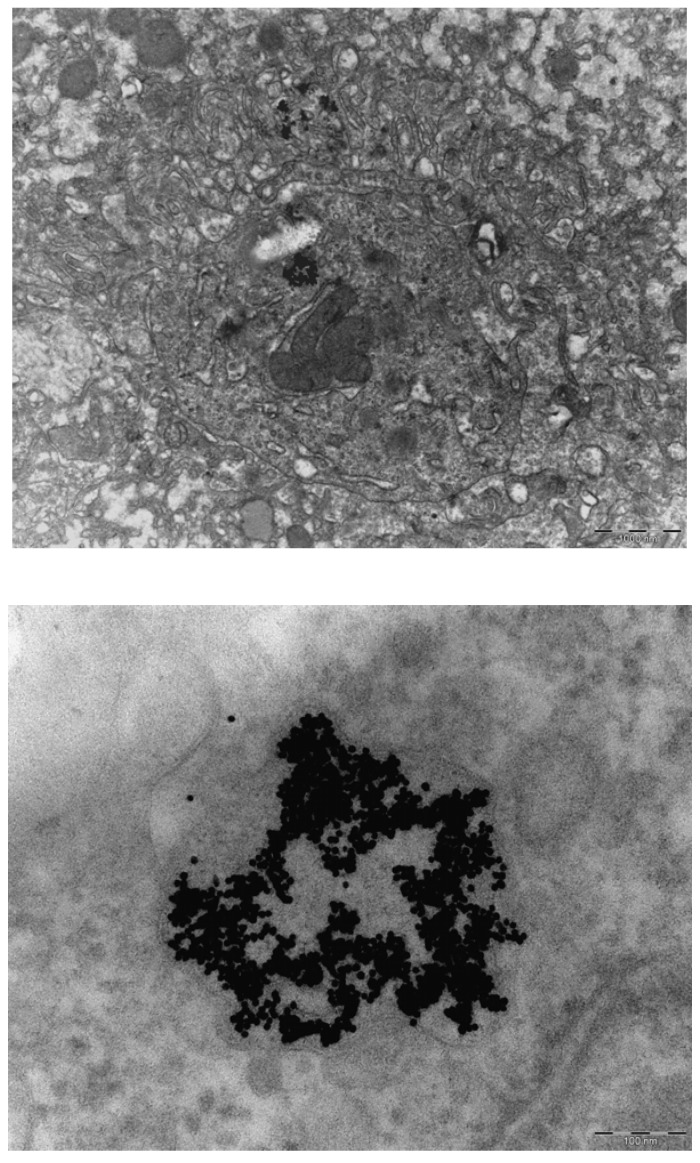
Presence of gold nanoparticles in Kupffer cells of the liver. Top. Presence of aggregates of 10 nm gold nanopartiocles in phago–lysosomes in Kupffer cells of the liver. The marker indicates 1000 nm. Bottom. Detail of nanoparticle aggregate in phago-lysosome. The marker indicates 100 nm.

**Figure 7:**
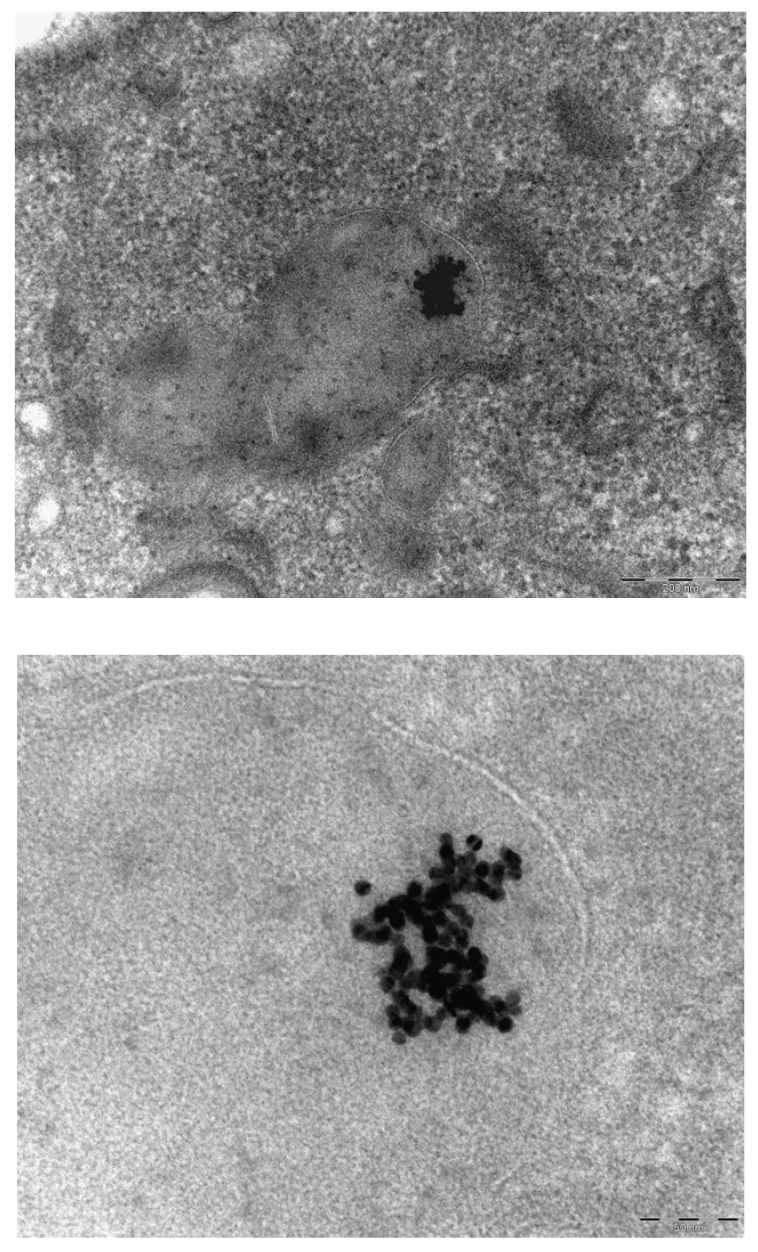
Presence of gold nanoparticles in macrophages of the spleen. Top. Presence of aggregate in macrophage of spleen. The marker indicates 200 nm. Bottom. Detail of aggregate. The marker indicates 50 nm.

In the liver of both animals injected with 10 nm gold nanoparticles, clusters of 10 nm particles were also observed in phago-lysosomes of Kupffer cells (Figure 6). Not all Kupffer cells contained particles, and the clusters of particles were not evenly distributed in the liver samples but the presence was restricted to certain areas. Also in the spleen of one of the two evaluated animals, similar clusters of particles were observed in macrophages but to a lesser extent (Figure 7). Only a few clusters were noted in the spleen. In both the liver and spleen the nanoparticles were present in phago-lysosomes. All other organs investigated, brain, heart and kidney were negative for the presence of the 10 nm gold nanoparticles.

In the animals treated with 250 nm gold particles, some dense particles (diameter 150–400 nm) were observed in the liver of both animals and the spleen of one animal (data not shown). In the other organs, no such dense particles were observed.

**Figure 8:**
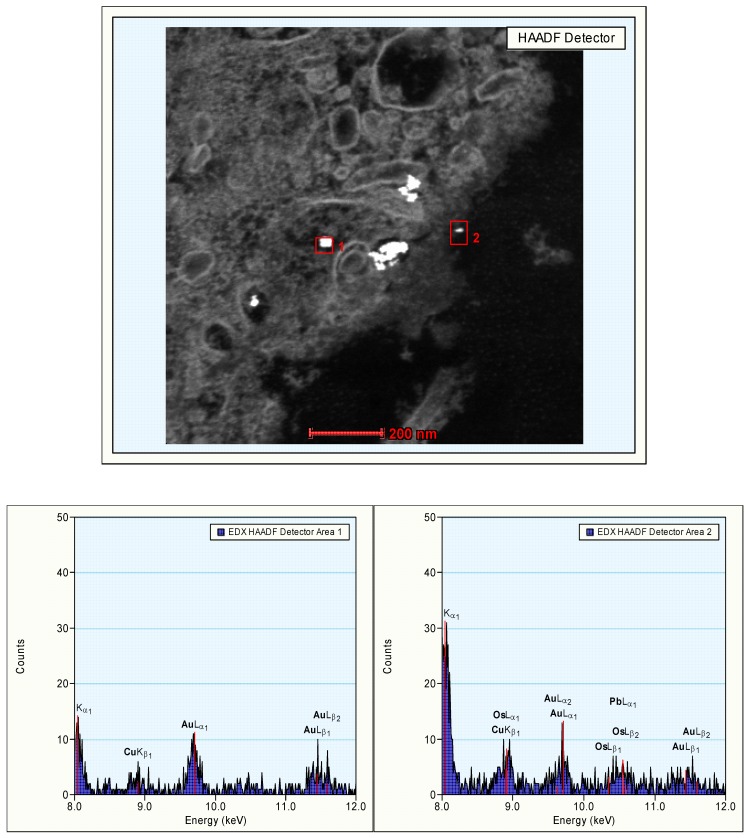
Presence of gold nanoparticles in Kupffer cells of the liver. Marker indicates 200 nm (top panel). Positive identification of the element gold in the observed nanostructures by EDX analysis. Area 1: a larger cluster of Au particles (10 nm; bottom left panel). Area 2: an area containing 2 Au particles (bottom right panel). In the surrounding areas, Os and Pb staining is present.

Additional analysis of the EDX spectrum was performed for positive identification of the observed nanostructures. It could be demonstrated that the clusters of 10 nm particles contained elemental gold and thus consisted of gold nanoparticles (Figure 8). 

## 3. Discussion and Conclusions

The TEM evaluation of the dispersion of gold nanoparticles that were administered revealed the presence of clusters of nanoparticles mainly in the dispersion of 10 nm gold nanoparticles. The arrangement of the nanoparticles in the dispersion indicated that the clusters were most likely agglomerates with weak binding forces. The identity of the gold nanoparticles could be confirmed by EDX evaluation. After intravenous administration, the gold nanoparticles may be covered with various proteins as present in blood such as serum albumin and apolipoproteins as demonstrated for polymeric nanoparticles [2]. These proteins on the nanoparticles may facilitate cellular uptake. In contrast, coating of nanomaterials with poly ethylene glycol (PEG) was demonstrated to limit cellular uptake and prolong the circulation time by inhibiting the interaction with proteins [3,4,5,6].

In our initial studies we observed 8–10 nm diameter structures in hepatocytes of the liver. These structures could not be identified by EDX spectrum analysis to contain gold. So, it was concluded that these structures were not the gold nanoparticles injected in the animals. We did not try to identify these structures. 

In our continued study brain, heart, kidney, liver and spleen were assessed for the presence of gold nanoparticles. Gold particles could be detected only in the liver and spleen, and only for the animals treated with 10 nm nanoparticles. Our results showed the presence of 10 nm particles in the phago–lysosomes of cells of the mononuclear phagocytic system (MPS) being macrophages in the spleen and the reliculo-endothelial system (RES) being the Kupffer cells in the liver. Both cell types have a function in clearing the blood from unwanted agents like bacteria and particulates. Although we did find the 10 nm particles in the liver, the nanoparticles were not evenly distributed in the liver tissue. There were local areas in which some nanoparticles and clusters of nanoparticles were observed while other areas were empty. These nanoparticle clusters in the cells were positively identified as gold nanoparticles by EDX spectrum analysis. To a lesser extent, similar nanoparticle clusters were observed in spleen macrophages. Whether these groups of nanoparticles are aggregates (with strong binding forces) or agglomerates (with weak binding forces) cannot be concluded from the TEM evaluation. The results of the TEM evaluation of the injected nanoparticle dispersions indicate that in the solution administered, the groups of nanoparticles are likely to consist of agglomerates. It is unknown whether the clusters found in the liver and spleen were taken up as clusters of nanoparticles or as single particles (or small clusters) ending up in the same cell. We did not perform a visual TEM analysis of blood samples in order to assess the physical shape of the nanoparticles in the circulation. Such an analysis is needed to assess whether the nanoparticles are present as single nanoparticles or as clusters of nanoparticles. Our results are in agreement with the results reported by Sadauskas *et al.* [7,8], who used autometallographic staining to detect 40 nm gold nanoparticles in the liver of mice. Due to the enhancing effect autometallography is suited to evaluate larger sections and thus larger tissue areas for the presence the nanoparticles when compared to EM sections for the presence the nanoparticles. After the initial uptake in the Kupffer cells of the liver, a gradual decrease over time was observed in the presence of the gold nanoparticles in the liver [7].

Particles are mainly taken up into cells by phagocytic pathways. Previously, the presence of different types of nanoparticles (gold and titanium oxide of 25 and 22 nm, respectively) was demonstrated inside red blood cells [9,10]. It was concluded that nanoparticles are able to cross the cell membrane by processes other than phagocytosis and endocytosis since erythrocytes do not have phagocytotic receptors. Diffusion, transmembrane channels, adhesive interactions, or other undefined transmembrane processes might play a role in this cellular uptake. Moreover, the uptake of ultrafine and 200 nm sized particles in macrophages was not blocked by the phagocytosis inhibitor cytochalasin D, whereas the uptake of 1,000 nm (1 mm) particles was inhibited [9], indicating the non-phagocytic nature of the uptake of the smaller nanoparticles. 

For the animals treated with 250 nm gold nanoparticles in our study, gold particles could not be detected in any of the tissue samples examined. 

The actual dose and the number of nanoparticles administered to the animals (see Table 1) was in total 5.1 × 10^12^ particles intravenously for the 10 nm size, while for the 250 nm 3.2 × 10^8^ particles were injected. Clusters of 10 nm gold particles were found in the liver but this was limited to certain areas of the liver. In the spleen it was much more difficult to find the 10 nm clusters, of which only a few could be found. It can be concluded that we have administered a relatively low dose to the animals. This explains why we did not find the 250 nm gold particles in the organs. Because of their size they are easier to find and to identify than the 10 nm particles. However, in view of the number of particles administered, the probability of finding these 250 nm particles in the ultra thin (80–100 nm) sections for TEM is rather low. For the 10 nm particles in the liver this would mean approximately 10^3^ particles in one TEM section (calculation, 10^12^ nanoparticles per liver of approximately 10 g, 10^11^ per gram liver, equals 10^11^ per cm^3^ liver; tissue section for TEM is 5 × 10^–5^ mm^3^ (1 mm × 0.5 mm × 0.1 µm); or 5 × 10^–8^ cm^3^ ) For 250 nm particles, the calculation would start at 10^8^ nanoparticles per liver resulting in 10^7^ per gram liver tissue, ending with less than 1 (10^–1^) nanoparticle per TEM section. This explains why we were unable to find the 250 nm particles in liver and spleen. In comparison, the ICP-MS method used for the detection of the element gold was thus very sensitive in detecting the gold nanoparticle distribution as even low percentages of the injected dose in various organs could be detected [1]. The liver and spleen contained the highest amount of the injected dose at 24 h after administration, up to 40% for the liver and 2% for the spleen.

In conclusion, we were only able to identify the organ localization for the 10 nm gold nanoparticles. Groups of 10 nm gold nanoparticles were present in phagocytozing cells of both the liver (Kupffer cells) and the spleen (macrophages). In the other organs investigated (brain, heart, kidney) no gold nanoparticles could be detected. Probably the dose administered was too low to demonstrate the presence of nanoparticles in tissue samples by TEM. Studies with a high concentration might enable observations on the localization of 250 nm nanoparticles inside or outside of cells. Our studies indicate that the *in vivo* identification of nanoparticles cannot only depend on the detection of nanosized structures in cells. An additional identification, for example by EDX (Energy Dispersive X-ray) detection of the composing elements, or a specific marker for the administered nanoparticles, may be necessary for a positive identification of the nanomaterial in tissues and cells.

**Table 1:** Gold Nanoparticle characteristics.

Particle size (diameter in nm)	10	50	100	250
Particle number/ml	5.7 × 10^12^	4.5 × 10^10^	5.6 × 10^9^	3.6 × 10^8^
Particle number/ml (x10^8^)	57000	450	56	3.6
Particle size (actual size in nm)	9.5	48.2	99.9	247.8
Surface area per particle (nm^2^)	283,5287	7298,674	31353,13	192909
Surface area per ml particles	1.62 × 10^15^	3.28 × 10^14^	1.79 × 10^14^	6.94 × 10^13^
Surface area per ml (× 10^13^)	162	32.8	17.9	6.9
Weight injected per animal (ng)	85,706	106,807	98,593	120,220
Number injected per animal^a^	5.1 × 10^12^	4.0 × 10^10^	5.0 × 10^9^	3.2 × 10^8^

^a^ The original solution was diluted 10% by adding 1 part of a 10 × concentrated PBS solution to 9 parts of the original solution.

## 3. Experimental Section

### 3.1. Animals

Male WU Wistar-derived rats, 6–8 weeks of age were obtained from the animal facility of the Institute (RIVM, Bilthoven, The Netherlands). Animals were bred under SPF conditions and barrier maintained during the experiment. Drinking water and conventional feed were provided *ad libitum*. Husbandry conditions were maintained according to all applicable provisions of the national laws, Experiments on Animals Decree and Experiments on Animals Act. The experiment was approved by an independent ethical committee prior to the study.

### 3.2. Experimental Design

Gold nanoparticles of 10, 50, 100 and 250 nm in aqueous suspension were obtained from SPI supplies, West Chester, PA, USA. The characteristics of the gold nanoparticles are presented in Table 1. The gold suspensions were 10% diluted by adding one part of 10-times concentrated phosphate buffered saline (10 × PBS) to nine parts of the gold suspension, in order to obtain a physiological solution for intravenous injection. Directly after PBS addition, the nanoparticle solutions of 10, 50 and 100 nm showed a change in color from red to blue indicating the formation of nanoparticle agglomerates/aggregates, whereas the 250 nm particles did not. The solutions remained clear and no sediments or agglomerates/aggregates were visually noted. A PBS control solution was prepared by adding one part of 10 × PBS to nine parts of distilled water (1:10). Treatment groups were as follows: 10 nm gold nanoparticles (n = 7), 50 nm gold nanoparticles (n = 2), 100 nm gold nanoparticles (n = 4), and 250 nm gold nanoparticles (n = 5), PBS control (n = 3). One milliliter of each freshly prepared solution was injected in the tail vein. The injections were well tolerated and no adverse effects were observed during the 24 h observation period. 

At 24 h after injection, blood and the following organs were collected: adrenals, aorta, brain, heart, kidney, liver, lung, lymph nodes (mesenteric and popliteal), spleen, testis, thymus, and vena cava. Organs were weighed, and tissue samples were homogenized and frozen for determination of gold content by inductively coupled plasma mass spectrometry (ICP-MS). EDTA blood was collected and stored in the refrigerator (4 °C). The results of the ICP-MS determinations of the gold content of blood and organs are reported elsewhere [1].

### 3.3. Transmission Electron Microscopy (TEM)

Samples of nanoparticle dispersions used for intravenous administration were prepared for evaluation by transmission electron microscopy. Samples were added to a carbon-coated formvar film, contrasted with 2% phosphotungsten acid pH 5.2, and allowed to dry. Without further preparation, the samples were evaluated by transmission electron microscopy. 

For TEM evaluation, from each group of animals treated with a different size of nanoparticles, tissue samples were collected from several animals. In this study various organs (brain, heart, kidney, liver and spleen) of two animals treated with 10 nm gold nanoparticles, and two animals treated with 250 nm gold nanoparticles were evaluated. 

Tissue samples were fixed in a mixture of 2% paraformaldehyde and 2.5% glutaraldehyde in 0.1 M sodium cacodylate buffer pH 7.4 for at least one week at +4 °C. Added to the buffer was 0.01 M CaCl_2_, 0.01 M MgCl_2_, and 0.1 M sucrose. After washing, a second fixation was done in sodium cacodylate buffer with 1% osmiumtetroxide and 1.5% potassiumhexanocyanoferrat. Tissue samples were dehydrated with serial alcohol and propylene oxide, impregnated and embedded in glycidether 100 (1,2,3-tris(2,3-epoxypropoxy)propan), which was polymerized at 60 °C. Semithin sections were prepared and stained with toluidin blue. Ultrathin sections of 50–70 nm were stained by uranyl acetate and lead citrate. Transmission electron microscopy (TEM) was performed using a FEI Company TECNAI 12 (FEI Company, Eindhoven, The Netherlands) transmission electron microscope. 

Additional studies at TEM level were performed for further identification of the presence of the element gold in various structures identified in the TEM evaluation as nanoparticles. These studies were performed using a TECNAI F30ST TEM operated at 300 kV. Mass sensitive HAADF (High Angular Annular Dark Field) detector was used for the detection of the presence of heavy (metallic) atoms in structures. The mass sensitive detection means that a higher brightness in the image corresponds to the presence of (a larger concentration of) heavier atoms. HAADF images are acquired in the scanning TEM mode. Using an Energy Dispersive X-ray (EDX) detector element characteristic X-rays were assessed. In the EDX spectrum, the detected signal is plotted as a function of the (characteristic) energy. Chemical compositions, in this case gold, can be obtained by quantification of the data.
